# Self-Reported Oral Health Among Elderly Immigrants Residing in Norway: A Cross-Sectional Study

**DOI:** 10.3390/ijerph22081292

**Published:** 2025-08-18

**Authors:** Wegdan Hasha, Stein Atle Lie, Anne N. Åstrøm, Manal Mustafa

**Affiliations:** 1 Oral Health Center of Expertise in Western Norway, P.O. Box 5867 Bergen, Norway; anne.aastrom@uib.no (A.N.Å.); manal.mustafa@vlfk.no (M.M.); 2 Department of Biomedicine, Faculty of Medicine, University of Bergen, P.O. Box 5009 Bergen, Norway; 3 Center for Translational Oral Research, Department of Clinical Dentistry, Faculty of Medicine, University of Bergen, P.O. Box 5009 Bergen, Norway; stein.lie@uib.no; 4 Department of Global Public Health and Primary Care, Faculty of Medicine, University of Bergen, P.O. Box 5009 Bergen, Norway

**Keywords:** oral health, elderly, immigrants, self-reported, Norway

## Abstract

Immigrants represent 20.8% of Norway’s population, with 22.2% aged 50+. This study aimed to assess oral health-related behaviors and perceived oral health in relation to sociodemographic factors among elderly immigrants aged 50+. Methods: 174 participants (94% response rate). Data on sociodemographic, oral hygiene, diet, snus use, xerostomia, and halitosis were collected using the self-reported questionnaire. *p*-value < 0.05 indicates statistical significance. Results: Participants’ mean age was 60.7, with 60% reporting poor self-perceived oral health, and over 64% had missing teeth. Participants reported poor oral hygiene habits (35.1%, *n* = 61), frequent sugar consumption (51.1%, *n* = 89), and smoking (16.1%, *n* = 28). Poor oral health was more common in those aged 60 and over (OR = 2.5, CI: 1.1–5.8) and with a narrow social network (OR = 3.8, CI: 1.7–8.5). Women had lower odds of sugar consumption (OR = 0.38, CI: 0.18–0.8) and smoking (OR = 0.27, CI: 0.09–0.7), but living in Norway for less than 3 years increased smoking odds (OR = 4.5, CI: 1.2–15.8). Moreover, being unmarried (OR = 5.5, *p* = 0.008), recent immigration (OR = 24.3, *p* < 001), and a narrow social network (OR = 4.7, *p* = 0.004) were associated with higher odds of missing teeth. Conclusions: Elderly immigrants in Norway reported poor oral health, tooth loss, and unhealthy dietary and hygiene habits, highlighting the need for targeted interventions.

## 1. Introduction

Global migration has transformed the demographics of many countries, resulting in culturally diverse societies [1]. In Norway, immigrants represent 20.8% of the total population, with 22.2% of immigrants aged 50 and older, according to Statistics Norway [2]. The combination of migration and an aging population presents a significant challenge for many countries in the twenty-first century [3]. Immigrants often encounter several difficulties in their new host country, regardless of the reason for their relocation. These challenges encompass economic difficulties, diminished retirement income resulting from changes in career trajectory, and obstacles related to language proficiency and cultural adaptation [4,5]. Furthermore, dietary habits typically shift due to various factors, including limited acculturation, the unavailability of familiar foods, and cultural influences on eating [6]. Notably, these dietary changes may lead to various health problems, including a higher risk of obesity, type 2 diabetes, cardiovascular diseases, and oral lesions such as caries and periodontitis [6,7].

The oral health status of senior adults with immigrant backgrounds is a growing concern in many high-income countries, including Norway [8]. Understanding their unique oral health challenges is crucial for public health planning and intervention as the immigrant population ages. A systematic review has shown that elderly immigrants residing in Europe often report poorer oral health compared to their native-born counterparts [9]. Social inequalities were demonstrated among Brazilian seniors, as those unable to see a dentist, nonwhites, and males reported a similar need for dental treatment to individuals using free oral healthcare services [10]. A comparative study of recently arrived adults from the Middle East and Africa in Norway showed that dental health was generally poor, with half of the study population reporting oral impacts on daily performance [11]. A previous study revealed that, unlike their Swedish counterparts, older immigrants were more likely to experience oral disorders and discomfort. They were also more likely to be edentulous, reported infrequent brushing, and used dental services less frequently [12]. Immigrants in both Norway and Sweden expressed less satisfaction with their dental health (64%) than their native-born counterparts (77.4%) [13].

Factors such as limited access to dental care, language barriers, and lower socioeconomic status contribute to these disparities. Additionally, cultural differences in dietary habits and oral hygiene practices can further impact oral health outcomes [14]. Education and place of origin were significant predictors of tooth loss in early-life social conditions [15]. A pilot study conducted among adult immigrants and asylum seekers in Finland revealed that they need health education as well as basic and urgent dental care [16]. Although there is growing interest in addressing oral healthcare inequities, recent studies conducted in New York City revealed a lack of information about oral health, access to dental care, and the utilization of dental services among older adult immigrants [14,16]. Investigations and research surveys are needed to investigate oral health problems and the management required.

Self-reporting of health conditions is increasingly used in oral epidemiological surveys to collect health data [17]. This approach is popular because it depends on individuals’ perceptions of their health and illness, is convenient, more affordable than clinical assessments, and can be easily integrated into health surveys [17,18]. Various sociodemographic factors can influence how individuals perceive their oral health, and self-reported oral health provides valuable insights into these perceptions [19]. The information gathered from the questionnaires about perceived oral health among immigrants will be valuable in assessing their oral status and dental healthcare needs. Moreover, the health assessments will enhance awareness and provide valuable intergenerational knowledge for immigrants from countries with different health concepts and fewer preventive measures.

Norway exhibited effective and high-quality medical care, supported by its health system; in contrast, high-cost adult dental care may require co-payment [20]. Furthermore, it is plausible that immigrants residing in Norway might have had limited exposure to the preventive oral health measures commonly implemented in Western Europe, which may lead to a pattern of dental service utilization primarily aimed at relieving acute oral problems [9,19]. While general health is routinely assessed for all newcomers to Norway, limited data on their oral health are available. Therefore, it is important to integrate dental status assessments into health evaluations to better identify and address care needs and to inform strategies to reduce oral health disparities in this population [20]. We tested the null hypothesis that there is no association between self-reported oral health and sociodemographic factors. The alternative hypothesis posited that such associations do exist. This study aimed to assess oral health-related behaviors and perceived oral health in relation to sociodemographic characteristics among elderly immigrants aged 50 and older.

## 2. Materials and Methods

### 2.1. Study Design and Setting

This cross-sectional study utilized a structured self-administered questionnaire to assess the association between perceived oral health and socio-demographic factors and to describe the frequency of oral health-related behaviors among immigrants residing in Bergen Municipality, Norway. Data collection began in October 2023 and concluded in October 2024. The manuscript was prepared in accordance with the STROBE (Strengthening the Reporting of Observational Studies in Epidemiology) checklist for cross-sectional studies (Supplementary File S1).

Participants aged 50 and older with immigrant backgrounds born outside Norway (Asia, Africa, and Eastern Europe) were invited to participate. Most participants were recruited through the Center for Migration Health (SEMI) in Bergen Municipality. Additionally, participants were recruited mainly from Bergen Municipality through various non-governmental organizations (NGOs), including The Church’s City Mission (Kirkens Bymisjon), Caritas Bergen, the Chinese church, the Somalian Mosque, and the Association for Somalis living in Bergen (TUSMO). Individuals who met the inclusion criteria were informed about the study, signed the consent form, and filled out the questionnaire (Supplementary File S2), with the help of researchers if needed. Participants were categorized into two age groups: 50–59 and 60 and above. The use of age 50 as the threshold for ‘elderly’ is supported by evidence that immigrants, particularly from low- and middle-income countries, often experience accelerated aging due to socioeconomic hardship, limited healthcare access, and migration-related stress [21].

Exclusion criteria included individuals who were unwilling to participate or unable to provide consent. All participants voluntarily contributed to the study, and written informed consent was obtained from each individual before participating under ethical standards.

Ethical approvals were granted by the Norwegian Agency for Shared Services in Education and Research (SIKT, 517864, 8 February 2023) and the Regional Committees for Medical and Health Research Ethics of Northern Norway (REK, 592684, 25 April 2023). Norwegian regulations ensured confidentiality throughout the research process.

### 2.2. Data Collection and Measures

The measurements of the variables are as follows:

1. Sociodemographic information was assessed in terms of age, sex, and country of origin, where it was categorized into Asia, Africa, and Eastern Europe. The ages of the study participants were dichotomized into two groups: 50–59 years old and ≥60 years old. The education variable was dichotomized into higher education (0), including university/higher qualification, and lower education (1), including primary school/secondary school/high school. Marital status was provided with options married (0) or single (1), and the occupation was dichotomized into either working (0), including (Student/working), or not working (1), which included (permission, joblessness, and retirement). The length of residence in Norway was dichotomized into long stay (0): ≥3 years and short stay (1): 1–2 years. Social networks were defined as broad (0)/narrow (1).

2. Oral health-related behaviors: This section includes two questions: “Are you a smoker?” and “Are you a snuff user?” with options (No (0)/Yes (1)) for both questions. Regarding oral hygiene behaviors, including brushing, dental floss use, and the use of mouthwash, the responses are presented as (1) Almost every day, (2) Once or twice per week, (3) Once or twice per month, (4) Rarely than once a month, and (5) Never. The sum scores of oral hygiene behaviors frequency (brushing, dental floss use, and mouthwash use) were computed, ranging from 3 to 15. Based on the mean score of 7, the oral hygiene behaviors were dichotomized into frequent (0) for scores ≤ 7, and infrequent (1) for scores > 7. Questions related to dietary habits include sugar in food and sugar in drinks: “How often do you usually eat sweets?” and “How often do you usually have drinks containing sugar, tea, coffee, cola, or soft drinks?” The response options are as follows: (1) Almost every day, (2) Once or twice per week, (3) Once or twice per month, (4) Rarely than once a month, and (5) Never. The sum scores of dietary habit frequency (sugar in food and sugar in drinks) were computed, ranging from 2 to 10. Based on the mean score of 4, the dietary habit is then dichotomized into infrequent (0) for scores > 4 and frequent (1) for scores ≤ 4. The dichotomization of oral health behaviors was based on standard recommendations [22,23].

3. Perceived oral health: The last five questions are as follows: The first question is, “How would you describe the condition of your mouth and teeth?” with the response items as follows: (1) Very good, (2) Good, (3) Neither good nor bad, (4) Bad, (5) Very bad. We dichotomized these items into two categories: good (0), which includes 1–2, and poor (1), which includes 3–5. The second question is “Status of your teeth” (present all my teeth (0)/missing many, all of my teeth (1)). The third and fourth questions are: “Have you ever had dry mouth?” and “Have you ever had problems with bad breath?” The options are either No (0) or Yes (1). The fifth question is “How are you satisfied or dissatisfied with the condition of your mouth and teeth?” with response options as follows: (1) Very satisfied, (2) Satisfied, (3) Neither satisfied nor dissatisfied, (4) Unsatisfied, and (5) Very unsatisfied. These items were dichotomized into ‘satisfied’ (0), including 1–2, and ‘not satisfied’ (1), including 3–5.

### 2.3. Statistical and Data Analysis

The statistical analysis was conducted using IBM-SPSS version 29 for Microsoft Windows. The significance level was set at 0.05, corresponding to a 95% confidence interval (CI).

The sample size was calculated based on a 5% level of confidence (yielding µ = 1.96), a margin of error of µ = 10%, and 23.5% of people over 65 were satisfied with their oral health [24]. We attributed a design effect of deff = 2.5 in the present formula.

$$n=\frac{\mathrm{deff}\;\mu \;2\;p(1-p)}{\varepsilon \;2}$$

Assuming a 10% dropout, we determined that the study would require a minimum of 172 participants.

Frequencies and percentages were used to describe the categorical variables. Odds ratios (OR), along with unadjusted and adjusted logistic regression analyses assessed the association between the dependent variables (oral health-related behaviors, self-reported oral health, teeth status, xerostomia, halitosis, and satisfaction with oral health) and the independent variables (age, sex, marital status, employment, education, residency in Norway, and social network). Based on previous research on similar topics, we have added the following covariates: sex, age, marital status, residency in Norway, education, work, and social network to minimize potential effects of confounders [25,26]. Also, the adjusted logistic regression analysis included independent variables that showed an association with the outcome variable in different prior unadjusted analyses.

## 3. Results

A total of 185 individuals were invited to participate, and 174 completed the survey, resulting in a response rate of 94.3%. Women represented 65.9% of the study population, and adults aged 50–59 were 50.3%. Most European participants (91.8%) had resided in Norway for 1–2 years. In contrast, most African participants and Asian participants (90.6%) had lived in Norway for three years or more. A narrow social network was more common among East European participants (50.0%). The additional distribution information of participants’ socio-demographics by continent of birth is shown in Table 1.

Table 2 presents the frequency distribution of the outcome variables, including oral health-related behaviors such as oral hygiene, sugar consumption, snuff, and smoking, perceived oral health indicators, including self-reported oral health, teeth status, xerostomia, halitosis, and satisfaction. Participants reported poor oral hygiene habits 35.1% (*n* = 61), frequent sugar consumption at 51.1% (*n* = 89), and smoking at 16.1% (*n* = 28). In this study, 60% (*n* = 105) reported poor oral health, and over 64% (*n* = 112) implied that they were missing many teeth. Xerostomia was reported in 29.9% (*n* = 52), while halitosis was found in 27% (*n* = 47). Regarding satisfaction with their oral health and teeth, 62.6% (*n* = 109) expressed dissatisfaction with their oral health and teeth.

Self-reported poor oral health among participants was associated with having a narrow social network, in both unadjusted (OR = 3.09, 95% CI 1.62–5.90, *p* ≤ 0.001) and adjusted (OR = 3.85, 95% CI 1.72–8.59, *p* = 0.001) models Table 3. Participants aged 60 years and over were 2.6 times more likely to report poor oral health than those aged 50–59 in both unadjusted (OR = 2.84, 95% CI 1.47–5.47, *p* = 0.002) and adjusted (OR = 2.59, 95% CI 1.14–5.86, *p* = 0.02). Only sex, marital status and employment status did not significantly impact self-reported oral health.

Table 4 presents the association between teeth status, reported as the presence of all teeth or missing many teeth, and sociodemographic variables. Females had a lower likelihood of missing many teeth compared to males in both unadjusted (OR = 0.45, 95% CI 0.22–0.91, *p* = 0.027) and adjusted models (OR = 0.12, 95% CI 0.35–0.42, *p* = 0.001). Unmarried individuals showed significantly higher odds of missing many teeth than married individuals (OR = 5.57, 95% CI 1.58–19.63, *p* = 0.008). Missing many teeth was strongly associated with living in Norway for less than three years compared to longer residency of over three years (OR = 24.37, 95% CI 6.90–86.00, *p* = 0.001), and having a narrow rather than a broad social network (OR = 4.71, 95% CI 1.63–13.60, *p* = 0.004). Age, education, and employment did not show any significant associations.

The result of this study indicates that participants who have lived in Norway for less than three years are more likely to have poor oral hygiene habits than those who have resided for over three years. This relationship remains strong in both the unadjusted (OR = 3.24, 95% CI 1.56–6.71, *p* = 0.002) and adjusted models (OR = 4.18, 95% CI 1.79–9.70, *p* ≤ 0.001). Sex, age, marital status, education, employment, and social networking showed no correlation with oral hygiene habits Table 5.

As represented in Table 6, females had lower odds of frequent sugar consumption compared to males in both unadjusted (OR = 0.46, 95% CI 0.24–0.88, *p* = 0.01) and adjusted models (OR = 0.38, 95% CI 0.18–0.81, *p* = 0.01). No significant associations were noted for age, marital status, residency in Norway, education, employment, and social networking.

Supplementary Table S1 presents the association between smoking and sociodemographic variables. Women showed lower odds of smoking compared to men in both unadjusted (OR = 0.30, 95% CI 0.13–0.69, *p* = 0.005) and adjusted models (OR = 0.27, 95% CI 0.09–0.74, *p* = 0.01). Smoking was strongly associated with living in Norway for less than three years compared to longer residency of over three years (OR = 4.53, 95% CI 1.29–15.82, *p* = 0.01), and having a narrow rather than a broad social network, indicated lower odds of smoking (OR = 0.26, 95% CI 0.09–0.73, *p* = 0.01). Age, marital status, education, and employment showed no significant associations with smoking habits.

Supplementary Table S2 indicated that people who have lived in Norway for less than three years had a higher likelihood of having halitosis (OR = 2.88, 95% CI 1.17–7.12, *p* = 0.02), while those who are 60 years and older reported a lower probability of having halitosis (OR = 0.42, 95% CI 0.18–0.95, *p* = 0.03). Sex, marital status, education, employment, and social networking were not correlated with halitosis. Xerostomia was not found to be associated with any sociodemographic factors.

The relationship between oral health satisfaction and sociodemographic factors is shown in Supplementary Table S3. Females have lower odds of being unsatisfied with their teeth, as shown in both unadjusted (OR = 0.39, 95% CI 0.19–0.81, *p* = 0.01) and adjusted models (OR = 0.34, 95% CI 0.14–0.81, *p* = 0.01). Participants who have lived in Norway for less than three years are 3.1 times more likely to be unsatisfied than those who have lived there for more than three years. This relationship remains strong in both the unadjusted (OR = 3.05, 95% CI 1.49–6.23, *p* = 0.002) and adjusted models (OR = 3.10, 95% CI 1.34–7.22, *p* = 0.008). No significant associations were noted for age, marital status, social network, education, or employment.

## 4. Discussion

The present study assessed self-reported oral health-related indicators and their association with sociodemographic factors among immigrants aged 50 and older in Norway. Participants from different continents contributed to the survey, with a predominant representation of females. A significant proportion of participants from Africa and Asia had resided in Norway for varying durations, while those from Eastern Europe reported shorter stays, ranging from one to two years. Approximately one-third of the participants retired, and over half had completed higher education. Dietary and hygiene habits vary between participants, with less sugar intake among females. More than 60% of the respondents reported missing many teeth, and almost one-third suffered from xerostomia. Though over 60% reported poor oral health. We found that having a broad social network, longer residency in Norway, and being 50 to 59 years old positively impact self-reported oral health. Therefore, the findings indicated rejection of the null hypothesis, where sociodemographic factors were found to impact the self-reported oral health.

In the present study, over 60% of participants reported tooth loss, with a notable association observed between missing many teeth and several sociodemographic factors. In contrast, a previous study conducted in Sweden among immigrants aged 60 and older found that more than 80% retained some of their natural teeth, with a mean presence of 22 teeth for females and 28 for males, as reported by the study authors [12]. It is important to note that the number of remaining teeth was not assessed in the present study, as participants only reported experiencing the loss of multiple teeth. We found that sex, marital status, length of residency, and a broad social network significantly influence tooth preservation. Although no association was found between tooth loss and educational level in this study, a contrasting finding was observed in a study conducted at Nord-Trøndelag among participants aged 68–77, showing a significant correlation between educational attainment and the number of remaining teeth. Specifically, individuals with higher levels of education were more likely to preserve more teeth [27].

In line with the current study, existing research indicates that women generally exhibit better oral health behaviors than men, including more frequent brushing, superior overall oral hygiene, and regular flossing [28,29]. 48.9% of participants in this study reported consuming high amounts of sugar with varying frequency. This finding corroborates the higher consumption of sugary beverages among elderly immigrants compared to their Norwegian counterparts in the same age group. Particularly, older individuals were more likely to consume coffee or tea with added sugar but less likely to consume sugar-sweetened drinks [28]. The Theory of Planned Behavior offers insight into the behavioral factors underlying poor self-reported oral health stated by participants. Frequent consumption of sugary foods and inadequate oral hygiene practices, particularly in environments where oral health is not prioritized, suggest low behavioral intentions shaped by negative attitudes, limited perceived control, and weak social norms reflected in their self-perceived poor oral health status [30]. In contrast to the present findings, previous research has indicated that acculturation tends to increase smoking rates among women while decreasing smoking rates among men [31]. Furthermore, the higher smoking rates observed among men in the current study align with previous data, where more than one-third of men worldwide smoke, compared to fewer smoking women [31,32]. Additionally, a higher prevalence of tobacco use has been observed among male immigrants (40%) compared to 15% of men born in Sweden. Other studies have reported a daily smoking prevalence of 15% among older people [32].

In the current study, the reported lower frequency of halitosis among the study participants might indicate more use of local dental care services and improved hygiene habits that positively enhance oral health over time. Notably, the higher prevalence of halitosis among adults aged 50–59 compared to those aged ≥60 in our study contrasts with some literature suggesting an increase in halitosis with age. Factors such as medication use, salivary flow rates, and oral hygiene practices might influence these findings [33]. Another possibility is bias, as admitting bad breath might not be socially accepted [34].

Consistent with the present work, a study conducted among adults in southern Halland, Sweden, found that xerostomia (mouth dryness) was notably more prevalent in older individuals [35]. Furthermore, in terms of sex differences, our findings contrast with previous research, reporting that xerostomia is more prevalent among women than men in the elderly Norwegian population [36]. An earlier report has found that self-reported dry mouth at ages 65 and 70 was more prevalent among participants with lower education levels and those who smoked daily [37]. Although the self-reported method used in the present work may not fully capture the comprehensive oral health status of immigrants, it remains a relatively simple and cost-effective approach to data collection. This method can facilitate implementation in large-scale survey studies. Additionally, by inquiring about participants’ daily routines, this approach may enhance their awareness of proper dietary and hygiene practices. Furthermore, self-reported oral health data can be correlated with other variables, such as social determinants of health and socioeconomic status, offering valuable insights into how these factors influence individuals’ perceptions of their oral health. Lastly, the findings, which include information on participants’ self-reports, will aid policymakers in gaining a more profound understanding of dental healthcare needs and might improve the oral health facilities for elderly immigrants.

The present study has several limitations. First, most participants were recruited in Bergen, which may not represent Norway’s immigrant community. Additionally, the recruitment strategy focused on participants engaged in planned social and cultural activities, which restricted the sample to a small subset of senior immigrants who attended these events and excluded those who did not participate. Although (65–70) is a common threshold for old age in Norway, we used age 50 due to growing evidence that immigrant populations experience accelerated aging [38]. This allowed us to better capture age-related health variations within this population, as supported by studies on premature chronic disease onset and lower life expectancy in migrant groups [21,38,39]. Another limitation of this study is that participants were only asked to report their biological sex, with the options ‘male’ and ‘female.’ Other gender identities were not included, which limits the inclusivity of our data and may not fully reflect the diversity of the study population. Furthermore, including immigrants with different social networks in Norway limited our classification to ‘broad’ and ‘narrow’ categories. This could be seen as a limitation, since social networks are a multidimensional concept, and splitting them into two groups might not fully capture their complexity [39]. Despite assurances of anonymity, the presence of the surveyor could have introduced response bias [34]. The reliability of the questionnaire was supported by the inclusion of items validated in previous studies, ensuring consistency with established measures. Furthermore, a pilot study was conducted to test the questionnaire for clarity, internal consistency, and ease of understanding. Feedback from the pilot phase was used to refine the instrument. Despite these steps, it is acknowledged that the use of self-reported oral health data may introduce variability due to differences in individual interpretation and self-assessment, which could affect the validity of the findings [40,41]. Furthermore, questions about dental health might not reflect the complete oral health status, as only tooth loss was considered, while periodontal status and carious lesions were not included in the present work.

## 5. Conclusions

This study highlights a high prevalence of tooth loss and poor self-rated oral health among senior immigrants in Norway, particularly among those with limited education, lower income, and restricted social networks. Participants with longer stays in Norway and more extensive social ties reported better oral health, suggesting that social inclusion may buffer against oral health decline. Furthermore, disparities in dietary and hygiene behaviors—such as frequent consumption of sugary foods and infrequent toothbrushing—emerged as modifiable risk factors. These insights point to the need for culturally tailored oral health programs that not only improve access to dental care but also promote healthier habits and community engagement among aging immigrant populations.

## Figures and Tables

**Table 1:** Socio-demographic distribution (%) of study participants by continent of birth.

		Asia *n* (%) 51 (29.3)	Africa *n* (%) 32 (18.4)	East Europe *n* (%) 87 (50.0)	Total *n* (%) 174
Sex	Male	22 (37.9)	7 (12.1)	29 (50.0)	58 (34.1)
	Female	29 (25.9)	25 (22.3)	58 (51.8)	112 (65.9)
Age	50–59 years old	23 (28.4)	18 (22.2)	40 (49.4)	81 (50.3)
	≥60 years old	22 (27.5)	14 (17.5)	44 (55.0)	80 (49.7)
Marital status	Married	28 (26.2)	17 (15.9)	62 (57.9)	107 (63.3)
	Unmarried	22 (35.5)	15 (24.2)	25 (40.3)	62 (37.6)
Education	High level	21 (27.6)	11 (14.5)	44 (57.9)	76 (45.5)
	Low level	30 (33.0)	18 (19.8)	43 (47.3)	91 (54.5)
Employment	Yes	23 (41.8)	9 (16.4)	23 (41.8)	55 (32.7)
	No	26 (23.0)	23 (20.4)	64 (56.6)	113 (67.3)
Residency i Noway	Long ≥3 years	37 (57.8)	21 (32.8)	6 (9.4)	64 (43.0)
	Short <3 years	7 (8.2)	0 (0.0)	78 (91.8)	85 (57.0)
Social network	Broad	17 (24.6)	17 (24.6)	35 (50.7)	69 (42.3)
	Narrow	34 (36.2)	13 (13.8)	47 (50.0)	94 (57.7)

**Table 2:** Frequency of outcome variables: oral health-related behaviors (oral hygiene, sugar consumption, use of snuff, smoking) and perceived oral health (self-reported oral health and satisfaction, teeth status, xerostomia, halitosis).

Variables		*n* (%)
	Oral hygiene habits	
Teeth brushing	Frequent	160 (92.5)
	Infrequent	13 (7.5)
Dental floss use	Frequent	78 (45.1)
	Infrequent	95 (54.9)
Mouthwash use	Frequent	46 (26.4)
	Infrequent	128 (73.6)
Summary of oral hygiene habits	Good	112 (64.4)
	Poor	61 (35.1)
	Sugar consumption	
Sugar in food	Frequent	45 (25.9)
	Infrequent	129 (74.1)
Sugar in drinks	Frequent	84 (48.3)
	Infrequent	90 (51.7)
Summary of sugar consumption	Frequent	89 (51.1)
	Infrequent	85 (48.9)
	Smoking and the use of snuff	
Smoking	Yes	28 (16.1)
	No	146 (83.9)
Snuff use	Yes	2 (1.1)
	No	172 (98.9)
	Perceived oral health	
Self-reported oral health	Good oral health	69 (39.7)
	Poor oral health	105 (60.3)
Teeth status	Present all teeth	62 (35.6)
	Missing many teeth	112 (64.4)
Xerostomia	Yes	52 (29.9)
	No	122 (70.1)
Halitosis	Yes	47 (27.0)
	No	125 (71.8)
Satisfaction	Satisfied	62 (35.6)
	Unsatisfied	109 (62.6)

**Table 3:** Unadjusted and adjusted odds ratios (OR) and 95% confidence intervals (CI) for the association between self-reported oral health and sociodemographic variables. The adjusted odds ratios (ORs) account for sex, age, marital status, education, employment, length of residence in Norway, and social network.

Predictors		Self-Reported Oral Health The Reference Category Is: Poor Oral Health					
		Unadjusted			Adjusted		
		OR	95% CI	*p*-Value	OR	95% CI	*p*-Value
Sex	Male	1					
	Female	0.64	0.33–1.24	0.19	0.58	0.25–1.36	0.21
Age	50–59 years old	1					
	≥60 years old	2.84	1.47–5.47	0.002	2.59	1.14–5.86	0.023
Marital status	Married	1					
	Unmarried	0.87	0.47–1.64	0.67	1.68	0.68–4.14	0.25
Residency in Norway	Long ≥ 3 years	1					
	Short < 3 years	2.18	1.10–4.29	0.025	2.14	0.91–5.03	0.08
Education	High level	1					
	Low level	1.89	1.02–3.53	0.04	1.59	0.69–3.67	0.27
Employment	Yes	1					
	No	1.69	0.88–3.22	0.11	0.65	0.26–1.60	0.34
Social Network	Broad	1					
	Narrow	3.09	1.62–5.90	<0.001	3.85	1.72–8.59	0.001

**Table 4:** Unadjusted and adjusted odds ratios (OR) and 95% confidence intervals (CI) for the association between teeth status and sociodemographic variables. The adjusted odds ratios (ORs) account for sex, age, marital status, education, employment, length of residence in Norway, and social network.

Predictors		Teeth Status (Present All Teeth/Missing Many Teeth) The Reference Category Is: Missing Many Teeth					
		Unadjusted			Adjusted		
		OR	95% CI	*p*-Value	OR	95% CI	*p*-Value
Sex	Male	1					
	Female	0.45	0.22–0.91	0.027	0.12	0.35–0.42	0.001
Age (years)	50–59 years old	1					
	≥60 years old	2.49	1.28–4.85	0.007	2.18	0.77–6.18	0.14
Marital status	Married	1					
	Unmarried	1.08	0.56–2.06	0.82	5.57	1.58–19.63	0.008
Residency in Norway	Long ≥ 3 years	1					
	Short < 3 years	9.85	4.23–22.95	<0.001	24.37	6.90–86.00	<0.001
Education	High level	1					
	Low level	0.96	0.51–1.81	0.91	0.97	0.33–2.84	0.96
Employment	Yes	1					
	No	1.92	0.99–3.69	0.05	0.92	0.31–2.78	0.88
Social Network	Broad	1					
	Narrow	2.47	1.28–4.73	0.006	4.70	1.63–13.60	0.004

**Table 5:** Unadjusted and adjusted odds ratios (OR) and 95% confidence intervals (CI) for the association between oral hygiene habits (teeth brushing, dental floss use, and mouthwash use) and sociodemographic variables. The adjusted odds ratios (ORs) account for sex, age, marital status, education, employment, length of residence in Norway, and social network.

Predictors		Oral Hygiene Habits The Reference Category Is: Poor Oral Hygiene Habits					
		Unadjusted			Adjusted		
		OR	95% CI	*p*-Value	OR	95% CI	*p*-Value
Sex	Male	1					
	Female	0.84	0.44–1.62	0.60	0.67	0.31–1.48	0.32
Age	50–59 years old	1					
	≥60 years old	1.48	0.78–2.83	0.23	1.19	0.55–2.59	0.65
Marital status	Married	1					
	Unmarried	1.46	0.77–2.78	0.24	2.06	0.90–4.69	0.08
Residency in Norwy	Long ≥ 3 years	1					
	Short < 3 years	3.24	1.56–6.71	0.002	4.18	1.79–9.70	<0.001
Education	High level	1					
	Low level	1.24	0.66–2.35	0.50	1.09	0.49–2.38	0.83
Employment	Yes	1					
	No	1.26	0.64–2.48	0.49	0.67	0.28–1.61	0.38
Social Network	Broad	1					
	Narrow	1.64	0.86–3.14	0.13	1.72	0.79–3.74	0.17

**Table 6:** Unadjusted and adjusted odds ratios (OR) and 95% confidence intervals (CI) for the association between sugar consumption (sugar in food and sugar in drinks) and sociodemographic variables. The adjusted odds ratios (ORs) account for sex, age, marital status, education, employment, length of residence in Norway, and social network.

Predictors		Sugar Consumption The Reference Category Is: Frequent Sugar Consumption					
		Unadjusted			Adjusted		
		OR	95% CI	*p*-Value	OR	95% CI	*p*-Value
Sex	Male	1					
	Female	0.46	0.24–0.88	0.01	0.38	0.18–0.81	0.01
Age	50–59 years old	1					
	≥60 years old	0.88	0.48–2.83	1.63	0.67	0.32–1.42	0.30
Marital status	Married	1					
	Unmarried	0.62	0.33–1.16	0.13	0.79	0.37–1.73	0.56
Residency in Norwy	Long ≥ 3 years	1					
	Short < 3 years	2.08	1.08–4.01	0.02	1.70	0.79–3.65	0.17
Education	High level	1					
	Low level	0.92	0.50–1.68	0.79	1.01	0.48–2.16	0.96
Employment	Yes	1					
	No	1.13	0.59–2.13	0.70	1.04	0.46–2.37	0.92
Social Network	Broad	1					
	Narrow	1.46	0.79–2.71	0.23	1.50	0.72–3.14	0.27

## Data Availability

Data are contained within the article and Supplementary Materials.

## Supplementary Materials

The following supporting information can be downloaded at https://www.mdpi.com/article/10.3390/ijerph22081292/s1, Table S1: Unadjusted and adjusted odds ratios (OR) and 95% confidence intervals (CI) for the association between smoking and sociodemographic variables. The adjusted odds ratios (ORs) account for sex, age, marital status, education, employment, length of residence in Norway, and social network. Table S2: Unadjusted and adjusted odds ratios (OR) and 95% confidence intervals (CI) for the association between halitosis and sociodemographic variables. The adjusted odds ratios (ORs) account for sex, age, marital status, education, employment, length of residence in Norway, and social network. Table S3: Unadjusted and adjusted odds ratios (OR) and 95% confidence intervals (CI) for the association between satisfaction with oral health and sociodemographic variables. The adjusted odds ratios (ORs) account for sex, age, marital status, education, employment, length of residence in Norway, and social networks. File S1: STROBE (Strengthening the Reporting of Observational Studies in Epidemiology) checklist for cross-sectional studies. File S2: Structured self-administered questionnaire to assess the association between perceived oral health and socio-demographic factors and to describe the frequency of oral health-related behaviors among immigrants residing in Bergen Municipality, Norway.

## Abbreviations

The following abbreviations are used in this manuscript:

SEMI	Center for Migration Health
NGOs	Non-governmental organizations
TUSMO	The Association for Somalis living in Bergen
