# Supplementary material for: Self-Reported Oral Health Among Elderly Immigrants Residing in Norway: A Cross-Sectional Study
Source: Int J Environ Res Public Health. 2025 Aug 18;22(8):1292. doi: 10.3390/ijerph22081292 (PMC12386976; doi:10.3390/ijerph22081292)
Supplement: Supplementary file 1 [file ijerph-22-01292-s001.zip › File S2.pdf]

Oral health, oral health-related quality of life and use of dental service among 50-70 years old with an immigrant background in Norway

The questionnaire is for you and contains questions about social issues, attitudes towards dental health and use of dental health services. Your participation is voluntary, and the answer will be anonymous. This will provide valuable information that can be used to improve communication, planning and implementation of dental health treatment among adults/elderly with an immigrant background. We ask you to answer all the questions and tick your answer option for each question.

Thank you for participating in this survey.

Some questions about social, dental health and treatment as you experience it.

1. Country of origin
2. Year of birth
3. How many years have you been living in Norway?
4. Gender

(1) ❍ Male

(2) ❍ Female

5. Marital status

(1) ❍ Married

(2) ❍ Single

6. Education

(1) ❍ Primary school

(2) ❍ Secondary school

(3) ❍ High school

(4) ❍ University

(5) ❍ Higher qualification

7. Your present work (you can cross more than one)

(1) ❍ Student

(2) ❍ Working

(3) ❍ In permission

(4) ❍ Jobbless

(5) ❍ Retired

8. Social network

(1) ❍ Restricted

(2) ❍ Wide network

9. Are you smoker?

(1) ❍ Yes

(2) ❍ No

10. Are you snus user?

(1) ❍ Yes

(2) ❍ No

11.How would you describe the condition of your general health?

(1) ❍ Very good

(2) ❍ Good

(3) ❍ Neither nor

(4) ❍ Bad

(5) ❍ Very bad

12. How often do you visit your medical doctor?

(1) ❍ Many times per year

(2) ❍ Twice a year

(3) ❍ Once yearly

(4) ❍ Rarely than once a year

(5) ❍ I haven't visited the medical doctor for the last 5 years

Oral health

1. How often do you usually brush your teeth?

(1) ❍ Almost every day

(2) ❍ once or twice per week

(3) ❍ once or twice per month

(4) ❍ Rarely than once a month

(5) ❍ Never

2. How often do you usually clean (floss) the space between your teeth?

(1) ❍ Almost every day

(2) ❍ once or twice per week

(3) ❍ once or twice per month

(4) ❍ Rarely than once a month

(5) ❍ Never

3. How often do you usually use the mouthwash?

(1) ❍ Almost every day

(2) ❍ once or twice per week

(3) ❍ once or twice per month

(4) ❍ Rarely than once a month

(5) ❍ Never

4. How often do you usually eat sweets?

(1) ❍ Almost every day

(2) ❍ once or twice per week

(3) ❍ once or twice per month

(4) ❍ Rarely than once a month

(5) ❍ Never

5. How often do you usually have drinks containing sugar (tea, coffee, cola, saft)?

(1) ❍ Almost every day

(2) ❍ once or twice per week

(3) ❍ once or twice per month

(4) ❍ Rarely than once a month

(5) ❍ Never

6. How would you describe the condition of your mouth and teeth?

(1) ❍ Very good

(2) ❍ Good

(3) ❍ Neither nor

(4) ❍ Bad

(5) ❍ Very bad

7. Status of your teeth

(1) ❍ Present all my teeth

(2) ❍ Missed many/all of my teeth

8. Have you ever had dry mouth?

(1) ❍ Yes

(2) ❍ No

9. Have you ever had problems with bad breath?

(1) ❍ Yes

(2) ❍ No

10. How are you satisfied or dissatisfied with the condition of your mouth and teeth?

(1) ❍ Very satisfied

(2) ❍ Satisfied

(3) ❍ Neither nor

(4) ❍ Unsatisfied

(5) ❍ Very unsatisfied

Use of dental health service

1. How often have you been to the dentist in the last 5 years?

(1) ❍ Many times, per year

(2) ❍ Twice a year

(3) ❍ Once yearly

(4) ❍ Rarely than once a year

(5) ❍ I haven't visited the dentist for the last 5 years

2. You always can get in contact with your dentist when there is need?

(1) ❍ Completely agree

(2) ❍ Agree

(3) ❍ Neither nor

(4) ❍ Disagree

(5) ❍ Completely disagree

3. You always get the dental treatment you need?

(1) ❍ Completely agree

(2) ❍ Agree

(3) ❍ Neither nor

(4) ❍ Disagree

(5) ❍ Completely disagree

4. How are you satisfied or dissatisfied with the dental treatment/follow up you received?

(1) ❍ Very satisfied

(2) ❍ Satisfied

(3) ❍ Neither nor

(4) ❍ Unsatisfied

(5) ❍ Very unsatisfied

5. When was your last dental visit?

(1) ❍ Not more than 6 months ago

(2) ❍ More than 6 months up to 1 year ago

(3) ❍ I cannot remember

6. What was the reason for your last dental visit?

(1) ❍ I had problem with my teeth

(2) ❍ Routine dental checkup

(3) ❍ Other reasons

7. Sometimes I cannot go to the dentist because of economic reasons?

(1) ❍ Completely agree

(2) ❍ Agree

(3) ❍ Neither nor

(4) ❍ Disagree

(5) ❍ Completely disagree

8. Maintaining teeth healthy is important for you?

(1) ❍ Completely agree

(2) ❍ Agree

(3) ❍ Neither nor

(4) ❍ Disagree

(5) ❍ Completely disagree

Oral Impacts on Daily Performance (OIDP)

During the past 6 months how often have problems with your mouth or teeth caused you any difficulty with the following situations:

1. Eating and enjoying food?

(1) ❍ Every day or nearly every day

(2) ❍ Once or twice a week

(3) ❍ Once or twice a month

(4) ❍ Rarely than once a month

(5) ❍ Never

2. Speaking and pronouncing clearly?

(1) ❍ Every day or nearly every day

(2) ❍ Once or twice a week

(3) ❍ Once or twice a month

(4) ❍ Rarely than once a month

(5) ❍ Never

3. Cleaning your teeth?

(1) ❍ Every day or nearly every day

(2) ❍ Once or twice a week

(3) ❍ Once or twice a month

(4) ❍ Rarely than once a month

(5) ❍ Never

4. Sleeping and relaxing?

(1) ❍ Every day or nearly every day

(2) ❍ Once or twice a week

(3) ❍ Once or twice a month

(4) ❍ Rarely than once a month

(5) ❍ Never

5. Smiling and showing teeth?

(1) ❍ Every day or nearly every day

(2) ❍ Once or twice a week

(3) ❍ Once or twice a month

(4) ❍ Rarely than once a month

(5) ❍ Never

6. Maintaining usual emotional status?

(1) ❍ Every day or nearly every day

(2) ❍ Once or twice a week

(3) ❍ Once or twice a month

(4) ❍ Rarely than once a month

(5) ❍ Never

7. Carrying out daily activity?

(1) ❍ Every day or nearly every day

(2) ❍ Once or twice a week

(3) ❍ Once or twice a month

(4) ❍ Rarely than once a month

(5) ❍ Never

8. Enjoying contact with people?

(1) ❍ Every day or nearly every day

(2) ❍ Once or twice a week

(3) ❍ Once or twice a month

(4) ❍ Rarely than once a month

(5) ❍ Never
