# Supplementary material for: Self-Reported Oral Health Among Elderly Immigrants Residing in Norway: A Cross-Sectional Study
Source: Int J Environ Res Public Health. 2025 Aug 18;22(8):1292. doi: 10.3390/ijerph22081292 (PMC12386976; doi:10.3390/ijerph22081292)
Supplement: Supplementary file 1 [file ijerph-22-01292-s001.zip › Supplementary Tables S1-S3.pdf]

Supplementary Table S1: Unadjusted and adjusted odds ratios (OR) and 95% confidence intervals (CI) for the association between smoking and sociodemographic variables. The adjusted odds ratios (ORs) account for sex, age, marital status, education, employment, length of residence in Norway, and social network.

**Predictors**		**Smoking**<br>The reference category is: Smoking					
		Unadjusted			Adjusted		
		OR	95% CI	P- value	OR	95% CI	P- value
Sex	Male	1					
	Female	0.30	0.13– 0.69	0.005	0.27	0.09 – 0.74	0.01
Age	50-59 years old	1					
	≥60 years old	0.65	0.28 – 1.49	0.31	0.62	0.23– 1.69	0.35
Marital status	Married	1					
	Unmarried	0.50	0.20 – 1.27	0.15	1.04	0.33 – 3.22	0.95
Residency in Norwy	Long ≥ 3 years	1					
	Short < 3 years	2.83	1.06– 7.56	0.03	4.53	1.29 – 15.82	0.01
Education	High level	1					
	Low level	1.14	0.50 – 2.59	0.75	1.41	0.52 – 3.83	0.49
Employment	Yes	1					
	No	2.01	0.77 – 5.28	0.16	1.61	0.50 – 5.17	0.42
Social Network	Broad	1					
	Narrow	0.56	0.24– 2.71	1.28	0.26	0.09 – 0.73	0.01

Supplementary Table S2: Unadjusted and adjusted odds ratios (OR) and 95% confidence intervals (CI) for the association between halitosis and sociodemographic variables. The adjusted odds ratios (ORs) account for sex, age, marital status, education, employment, length of residence in Norway, and social network.

Predictors	**Halitosis**<br>The reference category is: Presence of halitosis						
		Unadjusted			Adjusted		
		OR	95% CI	P-value	OR	95% CI	P-value
Sex	Male	1					
	Female	0.57	0.28 – 1.14	0.11	0.77	0.34– 1.71	0.51
Age	50-59 years old	1					
	≥60 years old	0.51	0.25 – 1.04	0.62	0.42	0.18– 0.95	0.037
Marital status	Married	1					
	Unmarried	0.26	0.11 – 0.59	0.001	0.46	0.18 – 1.16	0.09
Residency in Norway	Long ≥ 3 years	1					
	Short < 3 years	2.34	1.10 – 4.96	0.02	2.88	1.17 – 7.12	0.022
Education	High level	1					
	Low level	1.09	0.55 – 2.13	0.81	1.53	0.68 – 3.45	0.31
Employment	Yes	1					
	No	0.93	0.45 – 1.89	0.83	0.76	0.30 – 1.88	0.55
Social Network	Broad	1					
	Narrow	0.57	0.61– 2.46	0.11	1.12	0.49 – 2.53	0.79

Supplementary Table S3: Unadjusted and adjusted odds ratios (OR) and 95% confidence intervals (CI) for the association between satisfaction with oral health and sociodemographic variables. The adjusted odds ratios (ORs) account for sex, age, marital status, education, employment, length of residence in Norway, and social networks.

Predictors	**Satisfaction**<br>The reference category is: Unsatisfied						
		Unadjusted			Adjusted		
		OR	95% CI	P-value	OR	95% CI	P- value
Sex	Male	1					
	Female	0.39	0.19 – 0.81	0.01	0.34	0.14 – 0.81	0.015
Age	50-59 years old	1					
	≥60 years old	1.35	0.71 – 2.59	0.36	1.13	0.49 – 2.61	0.78
Marital status	Married	1					
	Unmarried	0.56	0.29 – 1.08	0.08	1.14	0.48 – 2.69	0.76
Residency in Norway	Long ≥ 3 years	1					
	Short < 3 years	3.05	1.49 – 6.23	0.002	3.10	1.34 – 7.22	0.008
Education	High level	1					
	Low level	1.40	0.74 – 2.64	0.29	1.29	0.56 – 3.01	0.55
Employment	Yes	1					
	No	1.69	0.87 – 3.27	0.12	0.93	0.37 – 2.31	0.87
Social Network	Broad	1					
	Narrow	1.81	0.95 – 3.47	0.07	1.54	0.69 – 3.42	0.29
